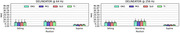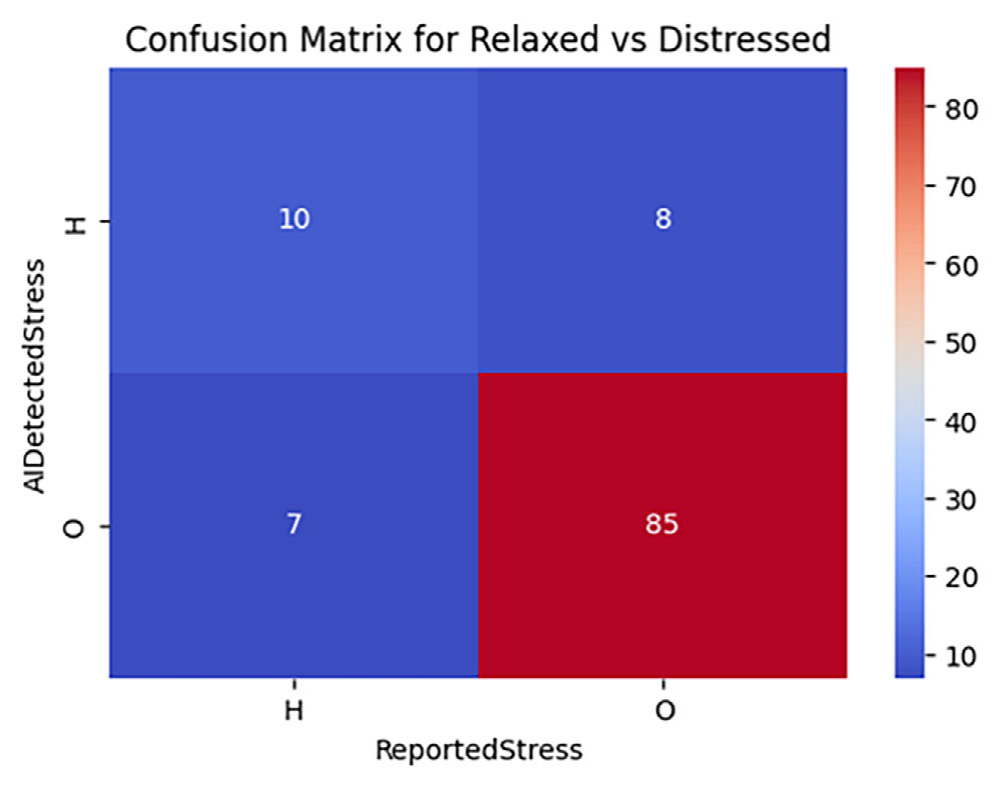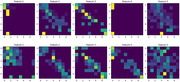# SmartSocks: a new data collection paradigm for dementia and other neurological disorders

**DOI:** 10.1002/alz.095065

**Published:** 2025-01-09

**Authors:** Zeke Steer, Prabha Thirthahalli Venkatesh, Elisa Mejia‐Mejia, William Wu Dennis, Patrick Ademola Ogundele, Annie Brooking, Iveta Eimontaite

**Affiliations:** ^1^ Milbotix Ltd, Chipping Norton, Oxfordshire United Kingdom; ^2^ Cranfield University, Bedford, Bedfordshire United Kingdom

## Abstract

**Background:**

Distress and agitation are predictors of entry into long‐term care and health inequalities (Schulz et al., 2004, Weir et al., 2022). Physiological data has been shown to reliably predict distress (Goodwin et al., 2019), yet wearable devices have low acceptance rates (Koumpouros & Kafazis, 2019). The current study discusses findings from a multifaceted approach investigating the detection of early signs of distress via physiological sensors in a foot‐worn device.

**Method:**

Firstly, the acceptance and concern ratings for a foot‐worn device, SmartSocks, wrist‐worn devices, Empatica E4 and Shimmer GSR+, and chest‐worn device, Equivital within a healthy population (N = 10) were assessed with a self‐report questionnaire. Secondly, data accuracy between Shimmer ECG and Polar OH1+ was compared within a healthy population (N = 12) in a standing, sitting and supine position. Finally, an ongoing ecologically valid feasibility trial (N = 2) involving participants with dementia or a learning disability is assessing the reliability of physiological data and AI‐detected stress from SmartSocks relative to subjective ratings of distress, the Abbey Pain Scale (APS), and the Neuropsychiatric Inventory (NPI).

**Result:**

Firstly, the SmartSocks received lowest concern ratings compared to wrist‐ and chest‐worn devices (1.64 vs <1.71). Secondly, the accuracy of SmartSocks pulse rate (PR) estimates obtained using photoplethysmography (PPG) in combination with the delineator algorithm was determined by comparing estimates to a Shimmer 1‐lead ECG, recording Mean Absolute Error (MAE)<5bpm at 64HZ for participants in a supine position (Fig. 1). This led to the development of new features for classifying PPG signal quality using neural networks, achieving approximately 95% accuracy. Finally, the initial stage of the feasibility trial indicated APS and NPI scores were lower after the participant with dementia wore SmartSocks for two weeks. Physiological data collected from the participant with a learning disability using SmartSocks showed moderate correlation (**χ^2^** = 0.45) between the reported and AI‐detected stress over the day (Fig. 2 & 3).

**Conclusion:**

Early findings suggest SmartSocks are more comfortable than comparable wrist‐ and chest‐worn devices, and validity of the data is comparable to other devices. Preliminary data obtained from people with dementia and learning disabilities suggest SmartSocks are capable of detecting distress to alleviate user discomfort.